# Second-year sentinel surveillance of bacterial meningitis in Jordan, Egypt, and Iraq: year 2 findings from the MenMap Network (2024–2025)

**DOI:** 10.1016/j.ijregi.2026.100984

**Published:** 2026-08-28

**Authors:** Lamia Al-Kershi, Zeina Abdel Majeed, Deema Al Bakri, Aseel Odeh, Magid Al Gunaid, Rund Al-Qaseer, Dominique A. Caugant, Muhamed-Kheir Taha, Saber Yezli, Areej Shoubaki, Marwa Abdel Shafy, Sahar Sami, Rawya Mohamed, Hussein Al Shammary, Ahmed Abdulsattar Salman, Florence Coste, Laure Badet, Amine Amiche, Ray Borrow

**Affiliations:** 1 Eastern Mediterranean Public Health Network, Amman, Jordan; 2 Meningococcal Reference Unit, UK Health Security Agency, Manchester Royal Infirmary, Manchester, UK; 3 Invasive Bacterial Infections Unit, Institut Pasteur, Paris, France; 4 Division for Infection Control, Norwegian Institute of Public Health, Oslo, Norway; 5 Innovation and Research, King Faisal Specialist Hospital and Research Centre, Riyadh, Saudi Arabia; 6 Sanofi Vaccines, Global Medical, Lyon, France; 7 Sanofi Vaccines, Global Medical, Dubai, United Arab Emirates; 8 Surveillance Department, Ministry of Health, Amman, Jordan; 9 Acute Infections Neurological Disease Control Program, Ministry of Health, Cairo, Egypt; 10 Central Public Health Laboratory, Ministry of Health, Cairo, Egypt; 11 Communicable Diseases Control Center, Ministry of Health, Baghdad, Iraq; 12 Central Public Health Laboratories, Ministry of Health, Baghdad, Iraq

**Keywords:** Bacterial meningitis, Eastern Mediterranean Region, Epidemiology, PCR, Public health, Surveillance

## Abstract

**Objectives:**

Bacterial meningitis remains a major cause of morbidity and mortality in low- and middle-income settings. The Meningitis and Septicemia Mapping Network (MenMap) was established to strengthen sentinel surveillance and molecular diagnostic capacity in the Eastern Mediterranean Region. This study presents results from the second year of MenMap surveillance under stabilized operational conditions.

**Methods:**

A prospective, hospital-based sentinel surveillance system was implemented across 15 referral hospitals in Jordan, Egypt, and Iraq between December 2024 and November 2025. Children aged 1 month to 18 years with suspected bacterial meningitis were enrolled. Clinical and laboratory data were collected, and specimens underwent real-time polymerase chain reaction. Descriptive analysis was performed.

**Results:**

A total of 1881 suspected cases were reported, of which 123 (6.5%) were laboratory-confirmed, with variation across countries (Egypt 10.5%; Jordan and Iraq 4.7%). *Streptococcus pneumoniae* was the predominant pathogen (87.0%), followed by *Neisseria meningitidis* (8.9%) and *Haemophilus influenzae* (4.1%). Confirmed cases were mainly observed among children aged 6-14 years and infants under 1 year. Ceftriaxone was the most administered antibiotic. Overall mortality was 5.7%.

**Conclusion:**

Under stabilized surveillance conditions, year 2 data provide stronger epidemiological estimates, confirming pneumococcal predominance while demonstrating improved laboratory performance, including 100% species-level concordance in annual external quality assessment, alongside persistent gaps in vaccination documentation.

## Highlights

• 1881 suspected bacterial meningitis cases were monitored across three countries in the Eastern Mediterranean Region.
• *Streptococcus pneumoniae* accounted for 87% of laboratory-confirmed infections.
• Standardized polymerase chain reaction surveillance improved diagnostic reliability across participating laboratories.
• Comparison with year 1 demonstrated stable pathogen distribution and enhanced laboratory performance.
• Findings support regional meningitis surveillance and evidence-informed vaccination strategies.

## Introduction

Meningitis remains a major global public health concern, causing substantial morbidity, mortality, and long-term disability across populations, with the greatest burden occurring in young children and in resource-limited settings. Among its different etiologies, bacterial meningitis is particularly important because of its potential for severe disease, death, and outbreaks. Despite substantial global reductions in incidence following the introduction of conjugate vaccines, invasive bacterial diseases caused by *Streptococcus pneumoniae, Neisseria meningitidis*, and *Haemophilus influenzae* continue to pose significant public health challenges, especially in low- and middle-income settings. In the Eastern Mediterranean Region (EMR), surveillance systems remain heterogeneous, with variable laboratory capacity, inconsistent case definitions, and limited access to molecular diagnostics. These gaps constrain accurate burden estimation and timely outbreak detection.

To address these challenges, the Meningitis and Septicemia Mapping Network (MenMap) was established to strengthen sentinel hospital-based surveillance and enhance real-time polymerase chain reaction (PCR) diagnostic capacity in Jordan, Egypt, and Iraq. The first year of MenMap implementation (December 2023-November 2024) demonstrated the feasibility of standardized multi-country surveillance and confirmed the predominance of *S. pneumoniae* among laboratory-confirmed cases, while highlighting persistent data gaps and operational challenges. That initial analysis primarily reflected system establishment and early implementation performance [1].

As an ongoing surveillance network, continued MenMap surveillance beyond the initial implementation year is important not only for identifying changes in disease epidemiology, but also for determining whether observed patterns persist over time and whether laboratory and surveillance performance can be maintained as the network matures. Longitudinal surveillance therefore provides an opportunity to assess the reproducibility of year 1 epidemiological findings, identify emerging changes in pathogen distribution, and evaluate the continuity and performance of the MenMap surveillance platform beyond its initial establishment phase.

Following this foundational phase, the second year of MenMap surveillance (December 2024-November 2025) represents the first full year of data generated under stabilized operational conditions, including harmonized laboratory workflows, continued annual external quality assessment (EQA), and strengthened data management procedures. Evaluating year 2 findings provides an opportunity to assess epidemiological consistency, pathogen distribution, and clinical patterns within a more mature surveillance framework, while contextualizing results relative to the initial implementation year.

### Study objectives

The primary objective of this study was to describe the epidemiology of suspected and laboratory-confirmed bacterial meningitis cases reported through the MenMap network during its second year of surveillance (December 2024-November 2025) in Jordan, Egypt, and Iraq.

Specifically, the study aimed to:

1. Characterize the distribution of confirmed pathogens, including species and meningococcal serogroups.
2. Describe demographic, seasonal, clinical, and outcome patterns among confirmed cases.
3. Contextualize year 2 findings in relation to those reported during the first year of MenMap surveillance.

## Methods

### Study design, setting, and case definitions

A prospective, hospital-based sentinel surveillance design was implemented across Jordan, Egypt, and Iraq from December 2024 through November 2025 under the MenMap framework. Because MenMap operates through selected sentinel hospitals rather than population-based surveillance, and defined catchment population denominators were not available for all participating sites, incidence rates could not be reliably estimated. The surveillance network included 15 sentinel hospitals (four in Jordan, four in Egypt, and seven in Iraq) selected in collaboration with national ministries of health to ensure geographic representation and laboratory referral capacity. Participating sites included different types of public hospitals, reflecting the organization of meningitis care and referral pathways within each country's health system. In Egypt, Hurghada and Sohag Fever Hospitals participated during the early phase of surveillance, while Alexandria and Minya Fever Hospitals joined the network during later implementation stages.

The overall surveillance structure and operational framework were established during the first year of implementation and have been described previously [1]. During year 2, surveillance activities continued under fully implemented standardized surveillance procedures, including harmonized case identification and reporting processes, standardized specimen handling and transport protocols, unified PCR workflows, and continued annual EQA participation across participating laboratories.

Children aged 1 month to 18 years presenting with suspected bacterial meningitis were eligible for inclusion. A suspected case was defined according to World Health Organization (WHO) guidelines [2] as acute onset of fever (≥ 38.0°C rectal or ≥ 37.5°C axillary) accompanied by at least one of the following: neck stiffness, altered consciousness, bulging fontanelle (in infants), new-onset seizures, severe headache, photophobia, or vomiting. Eligible cases were enrolled when cerebrospinal fluid (CSF) and/or blood specimens were collected for laboratory investigation and informed consent was obtained. Cases that did not fulfill these eligibility requirements were not enrolled in the surveillance.

### Surveillance procedures and data collection

At participating sentinel sites, trained clinicians identified suspected cases at presentation. Clinical and epidemiological data were recorded using a standardized MenMap case report form capturing demographics, symptoms, vaccination history (based on caregiver report and/or vaccination card when available), prior antibiotic use, and initial clinical management.

CSF specimens were obtained via lumbar puncture under aseptic conditions when clinically indicated. In cases where lumbar puncture was contraindicated or declined, blood samples were collected. Specimens were labeled with unique identifiers and transported under cold chain (2-8°C) to the respective Central Public Health Laboratories for molecular testing.

Standardized surveillance protocols, including case identification, data collection procedures, specimen handling, transport procedures, and reporting timelines, were maintained across all three countries to ensure comparability.

### Laboratory methods and quality assurance

#### Laboratory procedures

Real-time polymerase chain reaction (PCR) was used as the primary diagnostic method for pathogen detection. Molecular assays targeted conserved genes specific to the three principal bacterial pathogens: *lytA* for *S. pneumoniae, ctrA* and/or *sodC* for *N. meningitidis*, and *hpd* for *H. influenzae*.

PCR results were interpreted according to standardized cycle threshold (Ct) criteria defined within the MenMap protocol. Samples with Ct values ≤ 35 were considered positive; those between 36-40 were retested for confirmation; and values > 40 were considered negative. When feasible, conventional culture was performed according to the national laboratory capacity.

Laboratory workflows followed unidirectional processing to minimize contamination, with separate areas for nucleic acid extraction, reaction setup, and amplification.

#### EQA process

An EQA scheme was implemented in collaboration with a reference laboratory to monitor inter-laboratory performance. Participating laboratories received blinded panels to assess species-level detection accuracy and meningococcal serogroup identification. Performance feedback was provided, and corrective actions were undertaken when discrepancies were identified. Participating laboratories continued annual EQA participation throughout year 2 to ensure sustained diagnostic reliability and inter-laboratory consistency.

### Biosafety considerations

All laboratory activities were conducted in accordance with biosafety level 2 practices, including the use of personal protective equipment and certified Class II biosafety cabinets. Standard procedures for waste disposal and decontamination were followed at all participating laboratories.

### Data management and analysis

Data from case report forms and laboratory results were entered into secure national databases using unique identifiers to link clinical and molecular findings. De-identified datasets were centrally compiled for analysis.

Descriptive statistical analyses were performed to summarize case counts, pathogen distribution, demographic characteristics, seasonal patterns, clinical presentation, antibiotic administration, and outcomes. Categorical variables were reported as frequencies and proportions. No imputation was performed for missing data. Variables with missing information were analyzed using available-case analysis, and denominators are reported where they differ because of missing observations. Statistical analyses were conducted, and figures were generated using Microsoft Excel and R Programming Language (RStudio).

## Results

### Surveillance overview

Between December 2024 and November 2025, a total of 1881 suspected bacterial meningitis cases were reported across the 15 participating sentinel hospitals in Jordan, Egypt, and Iraq. Of these, 123 (6.5%) were laboratory-confirmed by PCR, 1733 (92.1%) tested negative, and PCR testing was not performed for 25 (1.3%) cases (Figure 1).

**Figure 1:**
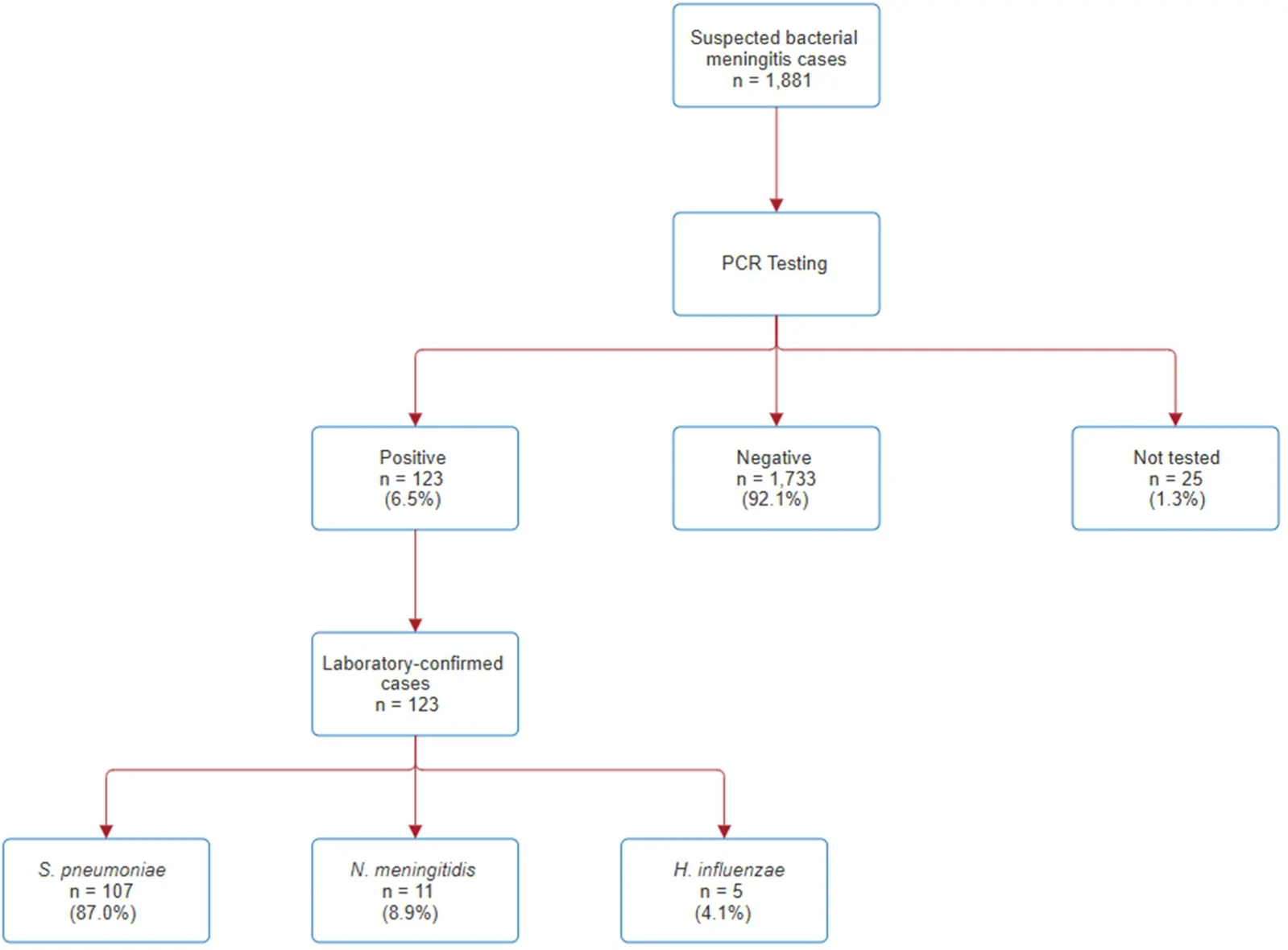
Flow of suspected bacterial meningitis cases through PCR testing and pathogen identification, MenMap year 2 (December 2024-November 2025). *H. influenzae, Haemophilus influenzae*; MenMap, Meningitis and Septicemia Mapping Network; *N. meningitidis, Neisseria meningitidis*; PCR, polymerase chain reaction; *S. pneumoniae, Streptococcus pneumoniae*.

### Country contribution

The distribution of suspected cases varied by country, with Iraq contributing the largest share (771 cases out of 1881), followed by Egypt (603 cases) and Jordan (507 cases). Of the 123 confirmed cases, 63 (51.2%) were reported in Egypt, accounting for over half of all confirmed cases during the study period, while Iraq and Jordan reported 36 (29.3%) and 24 (19.5%) confirmed cases, respectively.

Country-specific confirmation rates demonstrated notable variation. Egypt exhibited the highest proportion of confirmed cases relative to suspected cases (10.5%), whereas Jordan and Iraq showed similar confirmation rates (both approximately 4.7%). Across all countries, the majority of suspected cases tested negative for bacterial pathogens, and a small proportion did not have PCR testing performed (Figure 2).

**Figure 2:**
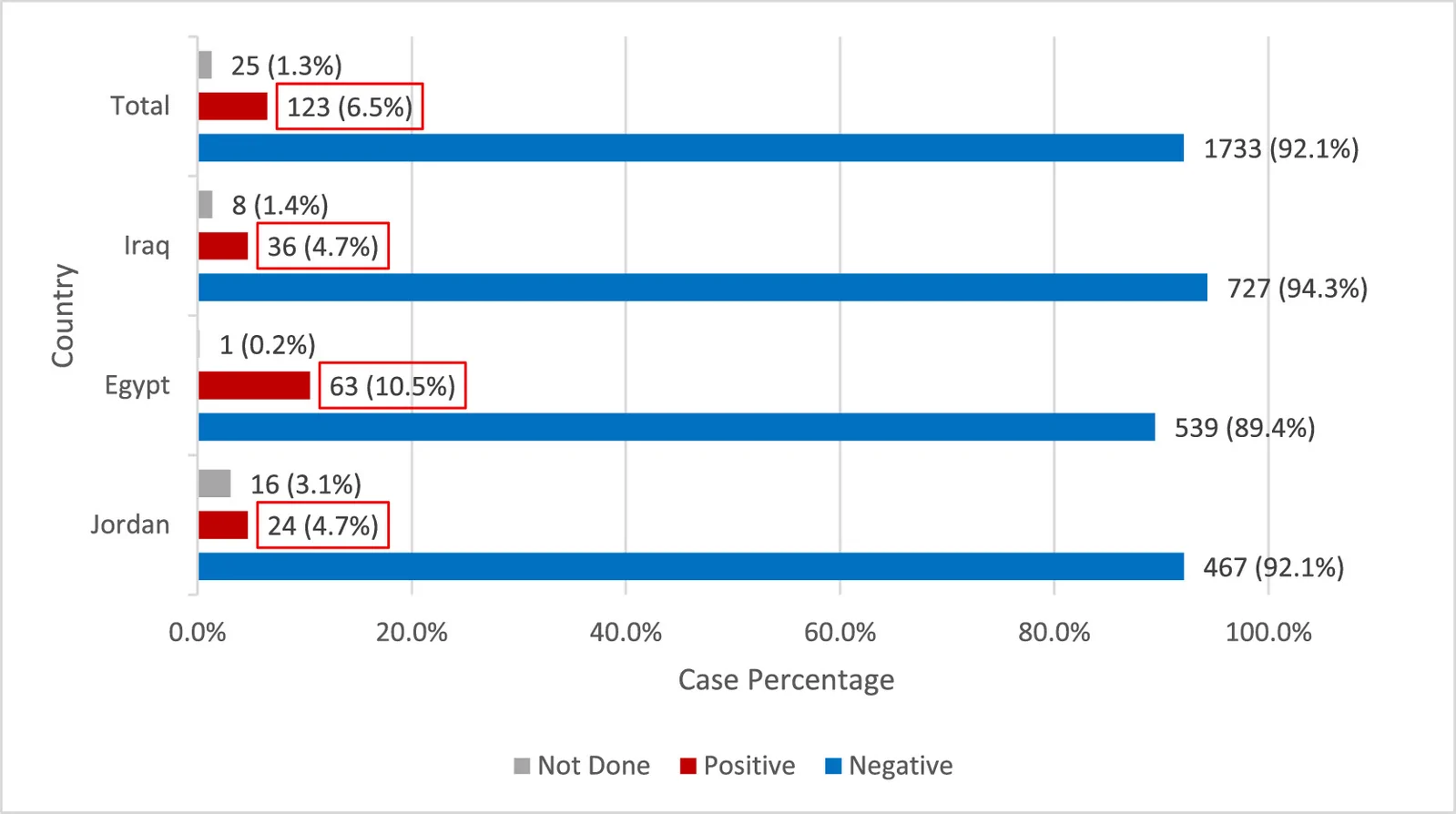
PCR testing outcomes among suspected bacterial meningitis cases by country, year 2 (December 2024-November 2025). PCR, polymerase chain reaction.

### Pathogen distribution

As shown in Figure 1, among the 123 laboratory-confirmed cases identified during year 2, *S. pneumoniae* was the predominant pathogen, accounting for 107 cases (87.0%). *N. meningitidis* represented 11 cases (8.9%), while *H. influenzae* accounted for five cases (4.1%).

Pathogen distribution showed consistent predominance of *S. pneumoniae* across all participating countries. In Iraq, all confirmed cases were attributable to *S. pneumoniae*. In Jordan and Egypt, *S. pneumoniae* remained the leading pathogen, with smaller proportions of *N. meningitidis* and *H. influenzae* detected.

Further characterization of *N. meningitidis* cases demonstrated heterogeneity in serogroup distribution. In Egypt, identified serogroups included serogroup B (*n* = 4), serogroup W (*n* = 2), and not serogrouped strains (*n* = 3). In Jordan, the serogroups of the detected cases were not identified.

Age-specific pathogen distribution is illustrated in Supplementary Figure S1. *S. pneumoniae* predominated across all age groups, while most *N. meningitidis* cases were observed among children aged 6-14 years.

### Demographic characteristics

Among the 123 laboratory-confirmed cases reported during year 2, males accounted for a slightly higher proportion of cases (57.7%) than females (42.3%).

Age distribution showed that confirmed cases were predominantly concentrated among children aged 6-14 years, representing the largest proportion (39.8%), followed by infants aged < 1 year (30.1%) and children aged 1-5 years (22.0%). Adolescents aged 15-18 years accounted for a smaller proportion of confirmed cases (8.1%).

Notable variation in age distribution was observed across countries. In Jordan, confirmed cases were primarily reported among infants aged < 1 year (54.2%), whereas in Egypt, the majority of cases occurred among children aged 6-14 years (50.8%) and adolescents aged 15-18 years (15.9%). In Iraq, confirmed cases were more evenly distributed across younger age groups, with children aged 1-5 years accounting for the largest proportion (41.7%), followed by infants (33.3%) and children aged 6-14 years (25.0%). No confirmed cases were reported among adolescents in Jordan or Iraq.

Distribution of confirmed cases across sentinel sites also varied within countries. In Jordan, Zarqa Hospital contributed the largest proportion of confirmed cases (33.3%), followed by Al Basheer Hospital (25.0%). In Egypt, Abbassia Fever Hospital accounted for the majority of confirmed cases (60.3%), while other sites contributed smaller proportions. In Iraq, Central Pediatric Hospital was the main contributor (44.4%), with remaining cases distributed across multiple pediatric hospitals (Table 1).

**Table 1:** Demographic characteristics of laboratory-confirmed bacterial meningitis cases by country, MenMap Year 2 (December 2024–November 2025).

Factor	Country							
	Regional		Jordan		Egypt		Iraq	
	N	%	n	%	n	%	n	%
**Gender**								
Female	52	42.3	11	45.8	30	57.7	11	30.6
Male	71	57.7	13	54.2	33	46.5	25	69.4
**Age Group**								
< 1 year	37	30.1	13	54.2	12	19.0	12	33.3
1- 5 years	27	22.0	3	12.5	9	14.3	15	41.7
6 - 14 years	49	39.8	8	33.3	32	50.8	9	25.0
15 - 18 years	10	8.1	0	0.0	10	15.9	0	0.0

Sentinel Sites^a^, ^b^			
Country	Hospital Name	N	%
**Jordan**	Al Basheer Hospital	6	25.0
	Al Karak Hospital	5	20.8
	Princess Rahma Pediatric Hospital	5	20.8
	Zarqa Hospital	8	33.3
**Egypt**	Abbasia Fever Hospital	38	60.3
	Alexandria Fever Hospital	6	9.5
	Hurghada Fever Hospital	1	1.6
	Minya Fever Hospital	5	7.9
	Sohag Fever Hospital	3	4.8
	Zagazig Fever Hospital	10	15.9
**Iraq**	Al-Zahraa Pediatrics Teaching Hospital	3	8.3
	Basra Pediatric hospital	1	2.8
	Central Pediatric Hospital	16	44.4
	Elwia Pediatric Hospital	5	13.9
	Ibn Al-Balady Pediatric Hospital	6	16.7
	Karbala Pediatric Hospital	5	13.9
	Kirkuk Pediatric Hospital	0	0.0

a Percentages reflect each hospital’s contribution to confirmed cases within the same country.

b For Egypt, Hurghada and Sohag Fever Hospitals were included in the surveillance network during the first quarter of the study period only and discontinued reporting thereafter. Alexandria and Minya Fever Hospitals joined the surveillance network starting from the second quarter of the study period.

### Seasonality and temporal patterns

The monthly distribution of laboratory-confirmed bacterial meningitis cases showed month-to-month variability across the surveillance period, as shown in Figure 3. At the regional level, confirmed cases fluctuated throughout the year, with relatively higher counts observed during March 2025 and October 2025, and lower counts reported during April-May and September 2025. However, no consistent temporal pattern was observed across the surveillance period.

**Figure 3:**
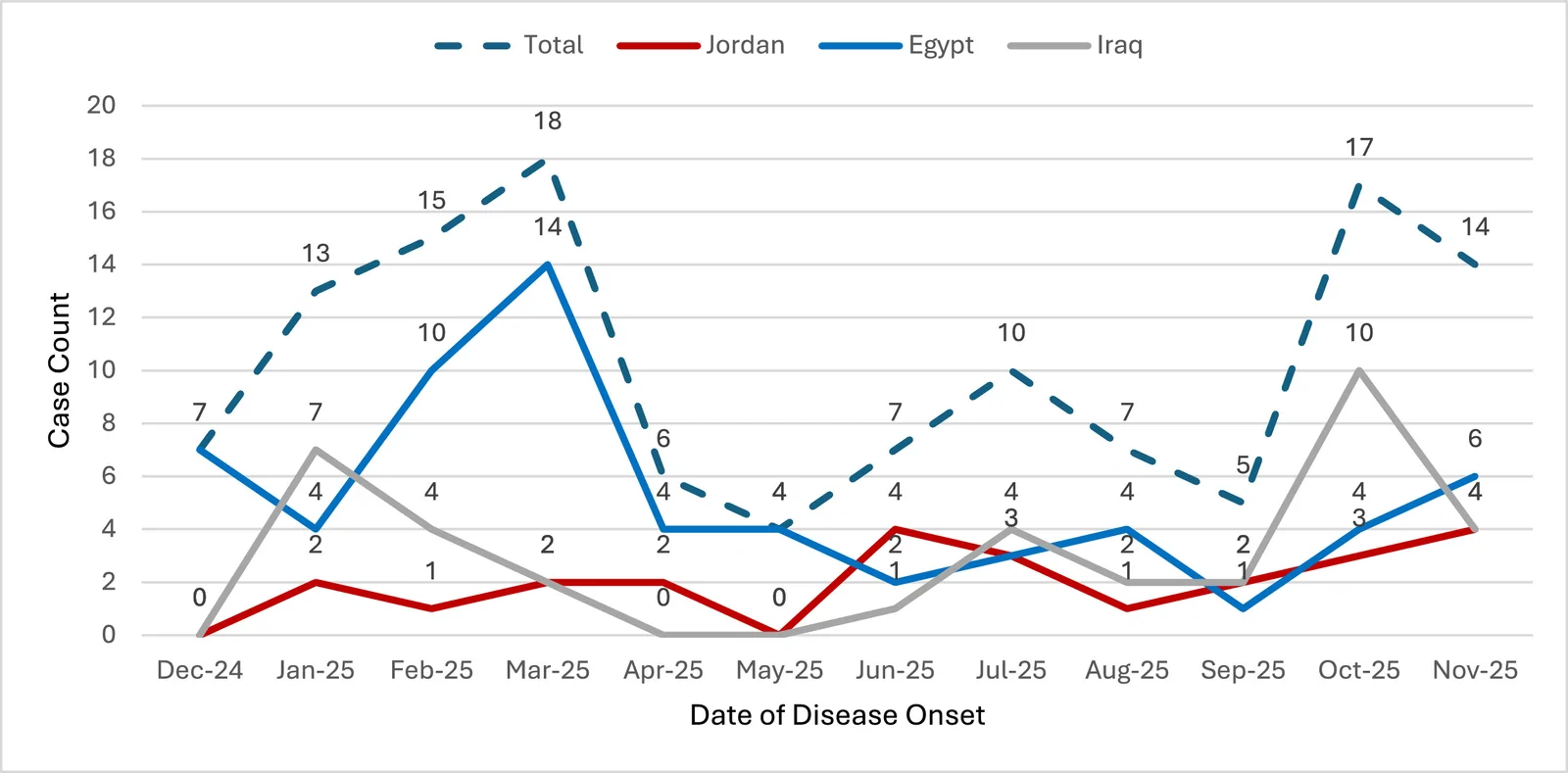
Monthly distribution of laboratory-confirmed bacterial meningitis cases by country and region, year 2 (December 2024-November 2025).

Country-specific patterns showed modest variation. In Jordan, confirmed cases remained low overall, with small increases observed in January, June, and November 2025. In Egypt, the highest number of confirmed cases was reported in March 2025, followed by relatively stable, low counts throughout the remainder of the year. In Iraq, confirmed cases were sporadic, with lower counts observed during March-May 2025, corresponding to a period when surveillance reporting was temporarily paused in the second quarter, and a subsequent increase was observed in October 2025 following the resumption of reporting.

The overall confirmed case counts remained low and did not demonstrate a sustained increasing or decreasing trend over time. No clusters of laboratory-confirmed cases or outbreaks were identified through the MenMap surveillance network during the year 2 surveillance period.

### Clinical presentation

Almost all confirmed cases met the clinical case definition, which includes fever as a required criterion (Supplementary Table S1). Fever was established based on measured temperature at presentation or a reported history of fever prior to presentation. In some cases, fever was not documented at the time of assessment, potentially reflecting self-medication with antipyretics before seeking care; therefore, health care providers also considered a patient- or caregiver-reported history of fever when assessing the case definition. Among additional reported symptoms, vomiting (56.1%), headache (42.3%), seizures (40.7%), and neck stiffness (34.1%) were the most commonly observed clinical features.

Neurological manifestations, including altered consciousness (26.0%) and lethargy (25.2%), were reported in approximately one-quarter of cases, while irritability was observed in 15.4%. Clinical signs suggestive of meningococcal infection, such as purpuric or hemorrhagic skin lesions, were identified in 11.4% of cases, whereas characteristic non-blanching purpura was rare (1.6%).

Other symptoms, including photophobia (10.6%) and toxic appearance (8.9%), were less frequently reported. Gastrointestinal symptoms such as nausea (8.1%) and diarrhea (8.1%), as well as non-specific symptoms including abdominal pain (5.7%) and chills (3.3%), were observed in a minority of cases. Severe clinical signs, including delayed capillary refill time (1.6%) and cold extremities (0.8%), were uncommon.

### Vaccination status

Vaccination status was analyzed among all suspected bacterial meningitis cases and stratified by PCR result (negative, positive, or not performed) (Supplementary Tables S2-S4). Overall, vaccination status was variably documented across the three countries, with a substantial proportion of cases classified as unknown, particularly for meningococcal and pneumococcal vaccines.

Among suspected cases in Jordan and Iraq, more than half of the individuals had unknown vaccination status for key bacterial meningitis vaccines, whereas Egypt demonstrated higher completeness of vaccination reporting. Overall, reported receipt of meningococcal and pneumococcal vaccines was low among suspected cases across the three countries; however, these findings should be interpreted in the context of differences in national immunization schedules and vaccine eligibility. In contrast, *H. influenzae* type b vaccination was more frequently reported, particularly in Egypt, where a higher proportion of suspected cases had documented *H. influenzae* type b vaccination compared with Jordan and Iraq. Across all countries, vaccination data were primarily based on caregiver reports, with limited availability of documented vaccination records.

To further explore the relationship between vaccination history and laboratory-confirmed infection, vaccination status against the corresponding pathogen was examined among laboratory-confirmed cases only (Supplementary Table S5). Documented vaccination was reported for three (2.8%) of 107 confirmed *S. pneumoniae* cases, two (40.0%) of five confirmed *H. influenzae* cases, and four (36.4%) of 11 confirmed *N. meningitidis* cases. Interpretation is limited by the small number of confirmed cases for some pathogens and the proportion of records with unknown vaccination status.

### Antibiotic administration

Broad-spectrum antibiotics were commonly used among laboratory-confirmed cases, with combination therapy reported for some patients. The overall treatment patterns were consistent with the use of broad-spectrum antibiotics commonly employed in the management of bacterial meningitis (Supplementary Table S6).

Ceftriaxone was the most frequently administered antibiotic across the three countries, reported in 89.4% of confirmed cases. Vancomycin was used in 23.6% of cases, while penicillin G was primarily reported in Iraq (47.2%) and was not used in Jordan or Egypt. Amoxicillin was infrequently administered (4.1%).

Other antibiotics were reported in a smaller proportion of cases (10.6%), including cefotaxime in Jordan and meropenem in Egypt and Iraq.

### Case outcomes

At the time of reporting, the most commonly recorded outcome among laboratory-confirmed cases was ongoing treatment, accounting for 51.2% of cases. Recovery was documented in 28.5% of cases, while 14.6% were referred for further clinical management, including transfer to specialized departments within the same hospital or to other health care facilities, such as referral from peripheral hospitals to central-level facilities (Supplementary Table S7).

Overall mortality among confirmed cases was 5.7%. Deaths were reported in Jordan and Egypt, whereas no fatalities were recorded in Iraq during the study period.

Outcome distribution varied across countries. In Egypt, over half of confirmed cases were reported as recovered (52.4%), while in Jordan and Iraq, most cases were still on treatment at the time of reporting. Referral of cases was more frequently observed in Iraq (41.7%) than in Jordan and Egypt.

Clinical outcomes stratified by pathogen are presented in Supplementary Table S8. Among the 107 laboratory-confirmed *S. pneumoniae* cases, 27 (25.2%) recovered, 55 (51.5%) remained on treatment at the time of reporting, 18 (16.8%) were referred, and 7 (6.5%) died. Among the 5 confirmed *H. influenzae* cases, 2 (40.0%) recovered, and 3 (60.0%) remained on treatment, with no reported deaths. Among the 11 confirmed *N. meningitidis* cases, 6 (54.5%) recovered, and 5 (45.5%) remained on treatment, with no deaths reported. All recorded deaths occurred among *S. pneumoniae* cases.

### EQA findings

EQA was conducted in 2025 in collaboration with the French National Reference Centre for Meningococci and *H. influenzae* at Institut Pasteur using an eight-sample non-culture panel distributed to participating laboratories. All laboratories achieved 100% concordance for species-level identification of the panel organisms, including *S. pneumoniae, N. meningitidis*, and *H. influenzae*. Reported Ct values were within ±2 standard deviations of the inter-laboratory mean for all samples, indicating consistent analytical performance across laboratories.

Compared with the previous EQA exercise conducted during year 1, species-level identification performance improved from 86% to 100% across participating MenMap laboratories. Performance in meningococcal genogrouping also improved, with Egypt increasing from 83% to 100%, Jordan from 50% to 66%, and Iraq from 80% to 100% (with *H. influenzae* serotyping samples excluded from the genogroup score calculation).

The EQA identified opportunities for further strengthening of *H. influenzae* characterization, particularly through implementation of PCR-based serotyping in laboratories where this capacity was not yet available. Overall, the findings demonstrated strong species-level diagnostic performance and improved inter-laboratory consistency compared with the previous assessment.

### Comparison between year 1 and year 2 surveillance findings

Comparison between the first and second years of MenMap surveillance demonstrated overall consistency in the epidemiological distribution of bacterial meningitis across participating countries (Figure 4). The total number of suspected cases decreased from 2104 in year 1 (December 2023-November 2024) to 1881 in year 2 (December 2024-November 2025), with a corresponding reduction in laboratory-confirmed cases from 191 to 123. Consequently, the overall PCR confirmation rate declined from 9.1% in year 1 to 6.5% in year 2.

**Figure 4:**
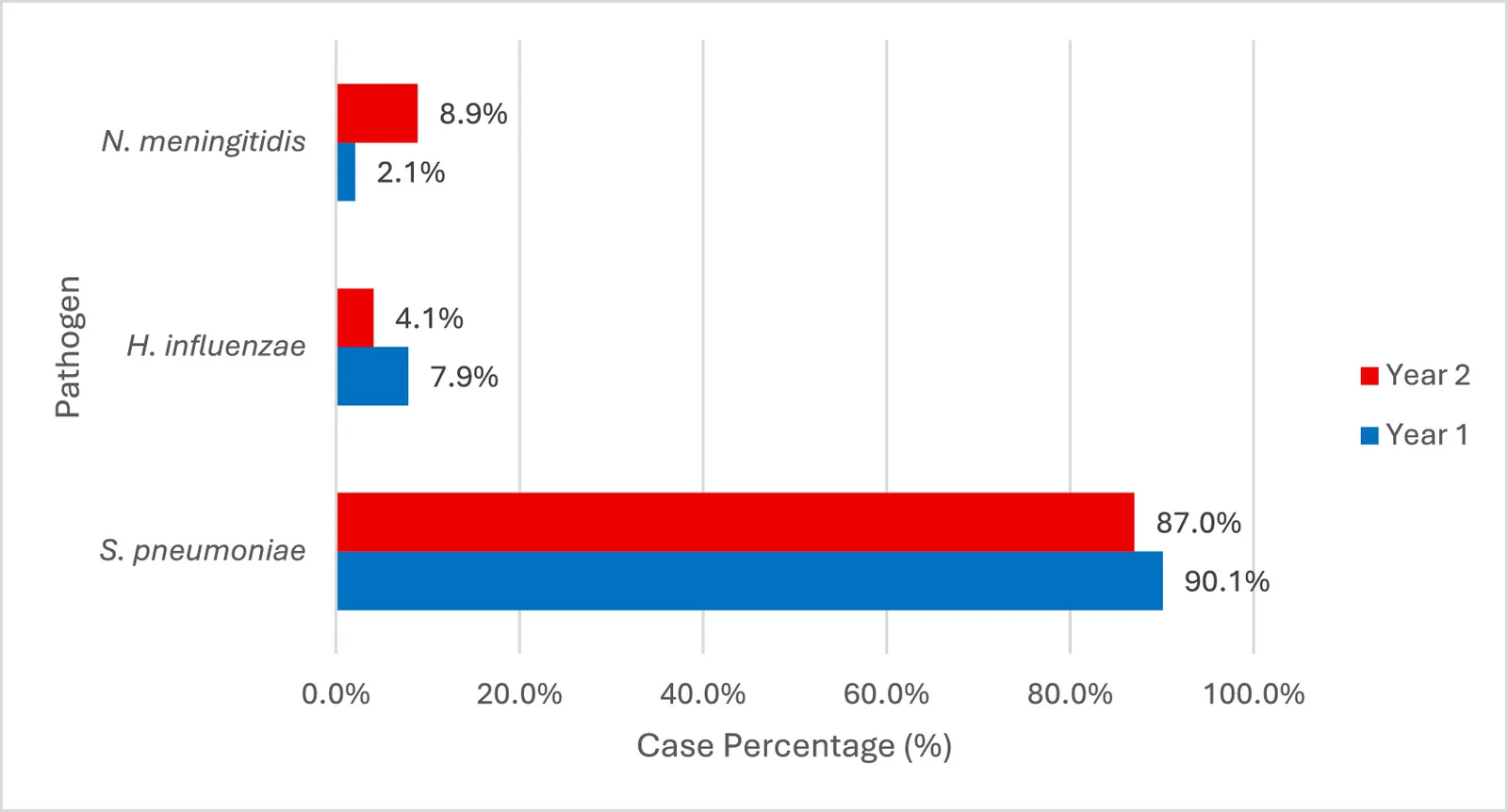
Distribution of laboratory-confirmed bacterial meningitis pathogens across year 1 and year 2 of MenMap surveillance. *H. influenzae, Haemophilus influenzae*; MenMap, Meningitis and Septicemia Mapping Network; *N. meningitidis, Neisseria meningitidis; S. pneumoniae, Streptococcus pneumoniae*.

Despite this reduction, pathogen distribution remained relatively stable across surveillance periods. *S. pneumoniae* continued to account for the majority of laboratory-confirmed cases, representing 90.1% of confirmed infections in year 1 and 87.0% in year 2. In contrast, the relative proportion of *N. meningitidis* increased from 2.1% to 8.9%, although absolute case numbers remained low, while *H. influenzae* accounted for a smaller proportion of confirmed cases in year 2. The emergence of confirmed meningococcal cases in Jordan during year 2 and the detection of serogroup W in Egypt highlight the value of sustained laboratory-based surveillance for monitoring evolving pathogen circulation patterns over time.

Similarly, demographic patterns continued to show a concentration of cases among younger age groups, with comparable clinical presentations across surveillance periods.

Notable differences were observed in surveillance performance indicators. The overall confirmation rate in year 2 was lower than that reported in year 1, which likely reflects improved sensitivity in case detection and broader inclusion of clinically suspected cases rather than a true decline in disease burden. In addition, laboratory performance demonstrated improvement, as evidenced by enhanced EQA outcomes and more consistent PCR results across participating laboratories.

Operational changes between the two years, including stabilization of surveillance procedures and expansion or adjustment of sentinel site participation in some countries, may also have contributed to observed differences in case distribution. Overall, the consistency in epidemiological patterns alongside improvements in system performance supports the reliability and maturity of the MenMap surveillance platform.

## Discussion

### Principal findings

This second-year analysis of the MenMap network provides insight into bacterial meningitis epidemiology under stabilized surveillance conditions across Jordan, Egypt, and Iraq. The findings confirm the sustained predominance of *S. pneumoniae* as the leading pathogen, with consistent distribution across countries and over time. Compared to year 1, the lower proportion of confirmed cases relative to suspected cases should be interpreted cautiously. As case definitions, laboratory protocols, and surveillance procedures remained largely unchanged between surveillance periods, the observed difference may reflect natural year-to-year variation, differences in case mix, or operational factors that were not fully captured within the surveillance dataset.

Despite the overall similarity in pathogen distribution between the two surveillance years, some differences were observed. The relative proportion of *N. meningitidis* increased in year 2, although the absolute number of cases remained low. Confirmed meningococcal cases were also identified in Jordan during year 2, and serogroup W was detected among cases in Egypt. These findings illustrate the value of continued laboratory-supported surveillance for monitoring changes in pathogen and serogroup circulation over time, even when the overall epidemiological pattern remains relatively stable.

The demographic and clinical patterns observed in year 2 remained broadly consistent with established epidemiology, with a substantial burden among children and classical clinical presentations predominating. Monthly fluctuations in confirmed cases were observed; however, no sustained temporal or seasonal trend was evident during the surveillance period, and no clusters of laboratory-confirmed cases or outbreaks were identified through the MenMap network. Interpretation of monthly patterns in Iraq should also take into account the temporary interruption in surveillance reporting during the second quarter, which may have influenced the observed distribution of cases over time.

Importantly, year 2 findings reflect improved surveillance performance and laboratory reliability. Enhanced EQA outcomes, consistent PCR performance, and more standardized data collection procedures indicate maturation of the MenMap system. At the same time, persistent gaps in vaccination documentation and variability in case follow-up highlight ongoing operational challenges that affect data completeness and interpretation. In particular, patient outcomes were recorded at the time of reporting rather than after completion of clinical management. Consequently, outcome categories such as “on treatment” and “referred” represent interim care status rather than final clinical outcomes and should be interpreted accordingly. As a result, the reported mortality should be interpreted cautiously, as deaths occurring after referral or among patients who were still receiving treatment at the time of reporting were not captured within the surveillance system.

### Interpretation in regional context

The predominance of *S. pneumoniae* observed in this study is consistent with global and regional evidence identifying pneumococcus as the leading cause of bacterial meningitis across multiple age groups, particularly in settings where pneumococcal conjugate vaccine coverage is incomplete or heterogeneous [3]. Large-scale analyses from the Global Burden of Disease study have demonstrated that *S. pneumoniae* contributes substantially to meningitis-related morbidity and mortality worldwide, particularly in low- and middle-income countries where disease burden remains highest [4].

The WHO Defeating Meningitis by 2030 roadmap similarly recognizes pneumococcus as one of the four principal pathogens responsible for the global burden of acute bacterial meningitis and highlights that the burden is disproportionately concentrated in resource-limited settings [5]. While the roadmap does not attribute this directly to a single factor, it emphasizes persistent gaps in vaccine introduction, suboptimal immunization coverage, and limitations in surveillance systems as key challenges affecting meningitis control globally.

In the EMR, variations in national immunization programs may partly explain the observed pathogen distribution. While some countries have introduced pneumococcal conjugate vaccines into routine childhood immunization schedules, others have yet to achieve widespread implementation or high coverage levels, contributing to continued transmission of pneumococcal disease [6]. The high proportion of cases among young children observed in this study is consistent with global epidemiological patterns, where children under five years bear the greatest burden of bacterial meningitis. WHO estimates indicate that approximately half of meningitis cases and deaths occur in this age group, reflecting both biological vulnerability and gaps in protective immunity in early life [5].

In addition, contextual factors such as population density, health care access, and mass gatherings may influence transmission dynamics in certain settings. For example, countries with frequent large-scale gatherings may experience increased circulation of respiratory pathogens, including meningitis-causing bacteria, although such associations are often multifactorial and require further investigation [7].

MenMap was established as an ongoing surveillance network, and longitudinal surveillance is necessary both to confirm the persistence of established epidemiological patterns and to identify changes that may emerge over time. Continued surveillance also provides an opportunity to monitor laboratory performance, detect changes in circulating pathogens and serogroups, and support timely identification of potential clusters or outbreaks. In parallel, ongoing efforts with national counterparts and partners aim to strengthen country ownership and institutionalization of surveillance activities within existing national systems, supporting the longer-term sustainability and expansion of the network.

The findings of this study are consistent with regional and global trends, reinforcing the need for strengthened surveillance systems, improved vaccine coverage, and sustained investment in laboratory capacity to better characterize and control bacterial meningitis in the EMR.

### Limitations

This study has some limitations that should be considered when interpreting the findings. Several data-related challenges observed during the first year of MenMap surveillance persisted into year 2. Vaccination history remained incompletely documented, with a substantial proportion of cases classified as “unknown,” largely due to reliance on caregiver recall and limited availability of written immunization records. Vaccination findings should also be interpreted in the context of differences in national immunization schedules and vaccine eligibility across participating countries, particularly as meningococcal vaccination is not universally included in routine childhood immunization programs. In addition, the small number of laboratory-confirmed cases for some pathogens and the substantial proportion of unknown vaccination histories limit interpretation of pathogen-specific vaccination findings.

In addition, outcome data were incomplete for a proportion of cases, as some patients were still on treatment at the time of reporting or were lost to follow-up following early discharge or referral to other facilities. Patient outcomes were recorded at the time of reporting rather than after completion of clinical management; therefore, categories such as “on treatment” and “referred” represent interim care status rather than final clinical outcomes. Consequently, the reported mortality may have been underestimated, because deaths occurring after referral or after the reporting period were not systematically captured.

Prior antibiotic use before health care presentation was not systematically captured and may have influenced laboratory confirmation rates. This remains a recognized challenge in many settings within the EMR, where antibiotics may be readily accessible before clinical evaluation, potentially reducing pathogen detection despite compatible clinical presentation [8]. Furthermore, pneumococcal serotyping was not performed, limiting the ability to assess circulating serotypes and their relevance to current vaccine formulations.

Operational challenges also affected data completeness during the study period. In Iraq, surveillance activities were temporarily disrupted during the second quarter, resulting in a pause in data reporting for several months before routine activities resumed.

Finally, MenMap is a sentinel hospital-based surveillance network and does not include all health care facilities within participating countries. Although sentinel sites were selected to provide geographic representation and laboratory referral capacity, the findings may not be fully representative of national disease patterns. Furthermore, because the surveillance network was based on selected sentinel hospitals and defined population denominators were not available for all participating sites, incidence rates could not be reliably estimated. Therefore, the results should be interpreted as reflecting trends within the surveillance network and should not be extrapolated to generate national incidence or burden estimates.

## Conclusion

The second year of MenMap surveillance provides robust evidence on the epidemiology of bacterial meningitis across Jordan, Egypt, and Iraq under stabilized operational conditions. The findings confirm the sustained predominance of *S. pneumoniae* and demonstrate consistent demographic and clinical patterns across the three countries.

Beyond epidemiological insights, year 2 results highlight the maturation of the MenMap surveillance system, reflected in improved laboratory performance, standardized data collection, and continued multi-country coordination. These advances support the reliability of the generated data and underscore the value of integrating molecular diagnostics into routine surveillance systems.

However, persistent gaps in vaccination documentation and incomplete outcome data indicate the need for strengthened data collection mechanisms. Enhancing linkage between surveillance and immunization systems, improving documentation of vaccination history, and ensuring follow-up of patient outcomes should be prioritized.

From a public health perspective, the continued predominance of pneumococcal disease reinforces the importance of expanding and optimizing pneumococcal vaccination strategies across the region. Strengthening laboratory capacity, sustaining external quality assurance, and maintaining coordinated regional surveillance efforts are essential to support early detection, guide policy decisions, and improve meningitis control in the EMR.

## CRediT authorship contribution statement

**Lamia Al-Kershi:** Writing – review & editing, Writing – original draft, Visualization, Formal analysis. **Zeina Abdel Majeed:** Writing – review & editing, Project administration. **Deema Al Bakri:** Writing – review & editing, Project administration. **Aseel Odeh:** Writing – review & editing. **Magid Al Gunaid:** Writing – review & editing, Supervision, Methodology, Conceptualization. **Rund Al-Qaseer:** Project administration. **Dominique A. Caugant:** Writing – review & editing, Validation, Supervision, Methodology. **Muhamed-Kheir Taha:** Writing – review & editing, Validation, Supervision, Methodology. **Saber Yezli:** Writing – review & editing, Validation, Supervision, Methodology. **Areej Shoubaki:** Investigation, Data curation. **Marwa Abdel Shafy:** Investigation, Data curation. **Sahar Sami:** Investigation, Data curation. **Rawya Mohamed:** Investigation, Data curation. **Hussein Al Shammary:** Investigation, Data curation. **Ahmed Abdulsattar Salman:** Investigation, Data curation. **Florence Coste:** Writing – review & editing, Supervision, Funding acquisition. **Laure Badet:** Project administration. **Amine Amiche:** Writing – review & editing, Supervision, Data curation. **Ray Borrow:** Writing – review & editing, Validation, Supervision, Methodology, Conceptualization.

## Declaration of competing interest

Magid Al Gunaid, Rund Al-Qaseer, Zeina Abdel Majeed, Deema Al Bakri, Aseel Odeh, and Lamia Al-Kershi are employed by GHD|EMPHNET, an organization that conducts epidemiological studies on infectious and non-infectious diseases, including those funded by Sanofi and other organizations, outside the scope of this work.

Muhamed-Kheir Taha performs contract work for the Institut Pasteur funded by GSK, Pfizer, and Sanofi and holds a patent (NZ630133A) with GSK titled "Vaccines for Serogroup X Meningococcus."

Ray Borrow conducts contract research on behalf of the UK Health Security Agency for GSK, PATH, Pfizer, and Sanofi.

Dominique A. Caugant and Saber Yezli have no conflicts of interest related to this project.

Amine Amiche, Florence Coste, Alp G. Dogu, and Laure Badet are employees of Sanofi.

## Appendix Supplementary materials

Image, application 1

Image, application 2

Image, application 3

Image, application 4

Image, application 5

Image, application 6

Image, application 7

Image, application 8

Image, application 9
