# Supplementary material for: Second-year sentinel surveillance of bacterial meningitis in Jordan, Egypt, and Iraq: year 2 findings from the MenMap Network (2024–2025)
Source: IJID Reg. 2026 Aug 28;21:100984. doi: 10.1016/j.ijregi.2026.100984 (PMC13672829; doi:10.1016/j.ijregi.2026.100984)
Supplement: Supplementary file 1 [file mmc1.docx]

Case Count

| Age Group | S. pneumoniae | H. influenzae | N. meningitidis |
| --- | --- | --- | --- |
| < 1 | 32 | 3 | 2 |
| 1 - 5 | 24 | 1 | 2 |
| 6 - 14 | 41 | 7 | 1 |
| 15 - 18 | 10 | 0 | 0 |

Figure S1: Distribution of laboratory-confirmed bacterial meningitis pathogens across age groups during Year 2 MenMap surveillance (December 2024–November 2025).
