# Supplementary material for: Second-year sentinel surveillance of bacterial meningitis in Jordan, Egypt, and Iraq: year 2 findings from the MenMap Network (2024–2025)
Source: IJID Reg. 2026 Aug 28;21:100984. doi: 10.1016/j.ijregi.2026.100984 (PMC13672829; doi:10.1016/j.ijregi.2026.100984)
Supplement: Supplementary file 2 [file mmc2.docx]

Table S1: Clinical outcomes of laboratory-confirmed bacterial meningitis cases by country, Year 2 (December 2024–November 2025)

| **Sign and Symptoms** | **Country** | | | | | | | |
| --- | --- | --- | --- | --- | --- | --- | --- | --- |
|  | **Regional** | | **Jordan** | | **Egypt** | | **Iraq** | |
|  | **N** | **%** | **n** | **%** | **n** | **%** | **n** | **%** |
| Fever | 117 | 95.1 | 24 | 100.0 | 61 | 96.8 | 32 | 88.9 |
| Vomiting | 69 | 56.1 | 16 | 66.7 | 38 | 60.3 | 15 | 41.7 |
| Headache | 52 | 42.3 | 9 | 37.5 | 34 | 54.0 | 9 | 25.0 |
| Seizures | 50 | 40.7 | 4 | 16.7 | 31 | 49.2 | 15 | 41.7 |
| Neck Stiffness | 42 | 34.1 | 3 | 12.5 | 29 | 46.0 | 10 | 27.8 |
| Altered consciousness | 32 | 26.0 | 4 | 16.7 | 28 | 44.4 | 0 | 0.0 |
| Lethargy | 31 | 25.2 | 14 | 58.3 | 11 | 17.5 | 6 | 16.7 |
| Irritability | 19 | 15.4 | 1 | 4.2 | 11 | 17.5 | 7 | 19.4 |
| Purpuric / hemorrhagic skin lesion* | 14 | 11.4 | 2 | 8.3 | 0 | 0.0 | 12 | 33.3 |
| Photophobia | 13 | 10.6 | 5 | 20.8 | 2 | 3.2 | 6 | 16.7 |
| Toxic appearance | 11 | 8.9 | 4 | 16.7 | 6 | 9.5 | 1 | 2.8 |
| Nausea* | 10 | 8.1 | 1 | 4.2 | 0 | 0.0 | 9 | 25.0 |
| Diarrhea* | 10 | 8.1 | 2 | 8.3 | 0 | 0.0 | 8 | 22.2 |
| Bulging fontanels | 10 | 8.1 | 1 | 4.2 | 5 | 7.9 | 4 | 11.1 |
| Leg pain | 9 | 7.3 | 0 | 0.0 | 6 | 9.5 | 3 | 8.3 |
| Abdominal pain | 7 | 5.7 | 1 | 4.2 | 2 | 3.2 | 4 | 11.1 |
| Chills | 4 | 3.3 | 0 | 0.0 | 3 | 4.8 | 1 | 2.8 |
| Characteristic purpura that does not blanch on pressure | 2 | 1.6 | 1 | 4.2 | 0 | 0.0 | 1 | 2.8 |
| Delay in capillary refill time | 2 | 1.6 | 2 | 8.3 | 0 | 0.0 | 0 | 0.0 |
| Cold extremities* | 1 | 0.8 | 1 | 4.2 | 0 | 0.0 | 0 | 0.0 |
| Arthralgia | 1 | 0.8 | 1 | 4.2 | 0 | 0.0 | 0 | 0.0 |
