# Supplementary material for: Second-year sentinel surveillance of bacterial meningitis in Jordan, Egypt, and Iraq: year 2 findings from the MenMap Network (2024–2025)
Source: IJID Reg. 2026 Aug 28;21:100984. doi: 10.1016/j.ijregi.2026.100984 (PMC13672829; doi:10.1016/j.ijregi.2026.100984)
Supplement: Supplementary file 3 [file mmc3.docx]

Table S2: Vaccination status by PCR result among suspected bacterial meningitis cases in Jordan, Year 2 (December 2024–November 2025)

| Vaccination Status | PCR Result | | | | | | | |
| --- | --- | --- | --- | --- | --- | --- | --- | --- |
|  | Negative | | Positive | | Not Done | | Total | |
|  | n | % | N | % | n | % | N | % |
| Ever Been Vaccinated to Bacterial Meningitis | 94 | 87.9 | 10 | 9.3 | 3 | 2.8 | 107 | 21.1 |
| **Meningococcal Vaccine** | | | | | | | | |
| No | 199 | 89.2 | 16 | 7.2 | 8 | 3.6 | 223 | 44.0 |
| Unknown ** | 268 | 94.4 | 8 | 2.8 | 8 | 2.8 | 284 | 56.0 |
| **Pneumococcal Conjugate Vaccine Type** | | | | | | | | |
| No | 193 | 89.4 | 16 | 7.4 | 7 | 3.2 | 216 | 42.6 |
| Unknown | 274 | 94.2 | 8 | 2.7 | 9 | 3.1 | 291 | 57.4 |
| ***Haemophilus Influenzae* type b** | | | | | | | | |
| Yes | 94 | 87.9 | 10 | 9.3 | 3 | 2.8 | 107 | 21.1 |
| No | 118 | 90.8 | 5 | 3.8 | 7 | 5.4 | 130 | 25.6 |
| Unknown ** | 255 | 94.4 | 9 | 3.3 | 6 | 2.2 | 270 | 53.3 |
| ** Unknown cases are derived from the self-reported information provided by the patients or their guardians. | | | | | | | | |
