# Supplementary material for: Second-year sentinel surveillance of bacterial meningitis in Jordan, Egypt, and Iraq: year 2 findings from the MenMap Network (2024–2025)
Source: IJID Reg. 2026 Aug 28;21:100984. doi: 10.1016/j.ijregi.2026.100984 (PMC13672829; doi:10.1016/j.ijregi.2026.100984)
Supplement: Supplementary file 4 [file mmc4.docx]

Table S3: Vaccination status by PCR result among suspected bacterial meningitis cases in Egypt, Year 2 (December 2024–November 2025)

| Vaccination Status | PCR Result | | | | | | | |
| --- | --- | --- | --- | --- | --- | --- | --- | --- |
|  | Negative | | Positive | | Not Done | | Total | |
|  | n | % | n | % | n | % | N | % |
| Ever Been Vaccinated to Bacterial Meningitis | 464 | 89.6 | 53 | 10.2 | 1 | 0.2 | 518 | 85.9 |
| **Meningococcal Vaccine** | | | | | | | | |
| Yes | 116 | 87.2 | 17 | 12.8 | 0 | 0.0 | 133 | 22.1 |
| *Polysaccharide* | *103* | *87.3* | *15* | *12.7* | *0* | *0.0* | *118* | *88.7* |
| *Conjugate* | *13* | *92.9* | *1* | *7.1* | *0* | *0.0* | *14* | *10.5* |
| *Not reported* | *0* | *0.0* | *1* | *100.0* | *0* | *0.0* | *1* | *0.8* |
| No | 351 | 90.7 | 35 | 9.0 | 1 | 0.3 | 387 | 64.2 |
| Unknown ** | 72 | 86.7 | 11 | 13.3 | 0 | 0.0 | 83 | 13.8 |
| **Pneumococcal Conjugate Vaccine Type** | | | | | | | | |
| No | 469 | 89.2 | 56 | 10.6 | 1 | 0.2 | 526 | 87.2 |
| Unknown | 70 | 90.9 | 7 | 9.1 | 0 | 0.0 | 77 | 12.8 |
| ***Haemophilus Influenzae* type b** | | | | | | | | |
| Yes | 348 | 90.4 | 36 | 9.4 | 1 | 0.3 | 385 | 63.8 |
| No | 120 | 87.0 | 18 | 13.0 | 0 | 0.0 | 138 | 22.9 |
| Unknown ** | 71 | 88.8 | 9 | 11.3 | 0 | 0.0 | 80 | 13.3 |
| ** Unknown cases are derived from the self-reported information provided by the patients or their guardians. | | | | | | | | |
