# Supplementary material for: Second-year sentinel surveillance of bacterial meningitis in Jordan, Egypt, and Iraq: year 2 findings from the MenMap Network (2024–2025)
Source: IJID Reg. 2026 Aug 28;21:100984. doi: 10.1016/j.ijregi.2026.100984 (PMC13672829; doi:10.1016/j.ijregi.2026.100984)
Supplement: Supplementary file 5 [file mmc5.docx]

Table S4: Vaccination status by PCR result among suspected bacterial meningitis cases in Iraq, Year 2 (December 2024–November 2025)

| Vaccination Status | PCR Result | | | | | | | |
| --- | --- | --- | --- | --- | --- | --- | --- | --- |
|  | Negative | | Positive | | Not Done | | Total | |
|  | n | % | N | % | n | % | N | % |
| Ever Been Vaccinated to Bacterial Meningitis | 270 | 93.1 | 14 | 4.8 | 6 | 2.1 | 290 |  |
| **Meningococcal Vaccine** | | | | | | | | |
| Yes | 66 | 93.0 | 3 | 4.2 | 2 | 2.8 | 72 | 9.2 |
| *MenA* | *1* | *100.0* | *0* | *0.0* | *0* | *0.0* | *1* | *1.4%* |
| *MenA+C* | *0* | *0.0* | *0* | *0.0* | *1* | *100.0* | *1* | *1.4%* |
| *MenACWY* | *57* | *93.5* | *3* | *4.9* | *1* | *1.6* | *61* | *84.7%* |
| *Not reported* | *9* | *100.0* | *0* | *0.0* | *0* | *0.0* | *9* | *12.5%* |
| No | 384 | 93.9 | 22 | 5.4 | 3 | 0.7 | 409 | 53.1 |
| Unknown ** | 277 | 95.2 | 11 | 3.8 | 3 | 1 | 291 | 37.8 |
| **Pneumococcal Vaccine Type** | | | | | | | | |
| Yes | 87 | 94.6 | 3 | 3.3 | 2 | 2.2 | 92 | 11.9 |
| *PCV10* | *6* | *85.7* | *0* | *0.0* | *1* | *14.3* | *7* | *7.6* |
| *PCV13* | *63* | *95.5* | *3* | *4.5* | *0* | *0.0* | *66* | *71.1* |
| *PPSV23* | *16* | *94.1* | *0* | *0.0* | *1* | *5.9* | *17* | *18.5* |
| *Not reported* | *2* | *100.0* | *0* | *0.0* | *0* | *0.0* | *2* | *2.2* |
| No | 353 | 93.1 | 23 | 6.1 | 3 | 0.8 | 379 | 49.2 |
| Unknown | 287 | 95.7 | 10 | 3.3 | 3 | 1 | 300 | 38.9 |
| ***Haemophilus Influenzae* type b** | | | | | | | | |
| Yes | 117 | 92.1 | 8 | 6.3 | 2 | 1.6 | 127 | 16.5 |
| No | 328 | 94.3 | 17 | 4.9 | 3 | 0.9 | 348 | 45.1 |
| Unknown ** | 282 | 95.3 | 11 | 3.7 | 3 | 1 | 296 | 38.4 |
| ** Unknown cases are derived from the self-reported information provided by the patients or their guardians. | | | | | | | | |
