# Supplementary material for: Second-year sentinel surveillance of bacterial meningitis in Jordan, Egypt, and Iraq: year 2 findings from the MenMap Network (2024–2025)
Source: IJID Reg. 2026 Aug 28;21:100984. doi: 10.1016/j.ijregi.2026.100984 (PMC13672829; doi:10.1016/j.ijregi.2026.100984)
Supplement: Supplementary file 6 [file mmc6.docx]

Table S5: Vaccination status against the corresponding pathogen among laboratory-confirmed cases, Year 2 (December 2024–November 2025)

| **Pathogen** | **Vaccination Status** | | | | | | | |
| --- | --- | --- | --- | --- | --- | --- | --- | --- |
|  | **Vaccinated** | | **Not Vaccinated** | | **Unknown** | | **Total** | |
|  | **n** | **%** | **n** | **%** | **n** | **%** | **N** | **%** |
| *S. pneumoniae* | 3 | 2.8 | 73 | 68.2 | 31 | 29.0 | 107 | 87.0 |
| *H. influenzae* | 2 | 40.0 | 1 | 20.0 | 2 | 40.0 | 5 | 4.1 |
| *N. meningitidis* | 4 | 36.4 | 5 | 45.5 | 2 | 18.2 | 11 | 8.9 |
