# Supplementary material for: Second-year sentinel surveillance of bacterial meningitis in Jordan, Egypt, and Iraq: year 2 findings from the MenMap Network (2024–2025)
Source: IJID Reg. 2026 Aug 28;21:100984. doi: 10.1016/j.ijregi.2026.100984 (PMC13672829; doi:10.1016/j.ijregi.2026.100984)
Supplement: Supplementary file 7 [file mmc7.docx]

Table S6: Antibiotic administration among laboratory-confirmed bacterial meningitis cases by country, Year 2 (December 2024–November 2025)

| **Antibiotic Type** | **Country** | | | | | | | |
| --- | --- | --- | --- | --- | --- | --- | --- | --- |
|  | **Regional** | | **Jordan** | | **Egypt** | | **Iraq** | |
|  | **N** | **%** | **n** | **%** | **n** | **%** | **n** | **%** |
| Amoxicillin | 5 | 4.1 | 4 | 16.7 | 0 | 0.0 | 1 | 2.8 |
| Ceftriaxone | 110 | 89.4 | 17 | 70.8 | 60 | 95.2 | 33 | 91.7 |
| Penicillin G | 17 | 13.8 | 0 | 0.0 | 0 | 0.0 | 17 | 47.2 |
| Vancomycin | 29 | 23.6 | 3 | 12.5 | 15 | 23.8 | 11 | 30.6 |
| Other* | 13 | 10.6 | 7 | 29.2 | 3 | 4.8 | 3 | 8.3 |
| *This category groups less frequently reported antibiotics that were not part of the main list. | | | | | | | | |
