# Supplementary material for: Second-year sentinel surveillance of bacterial meningitis in Jordan, Egypt, and Iraq: year 2 findings from the MenMap Network (2024–2025)
Source: IJID Reg. 2026 Aug 28;21:100984. doi: 10.1016/j.ijregi.2026.100984 (PMC13672829; doi:10.1016/j.ijregi.2026.100984)
Supplement: Supplementary file 8 [file mmc8.docx]

Table S7: Clinical outcomes of laboratory-confirmed bacterial meningitis cases by country, Year 2 (December 2024–November 2025)

| **Case Outcome** | **Country** | | | | | | | |
| --- | --- | --- | --- | --- | --- | --- | --- | --- |
|  | **Reginal** | | **Jordan** | | **Egypt** | | **Iraq** | |
|  | **N** | **%** | **n** | **%** | **n** | **%** | **n** | **%** |
| Recovered | 35 | 28.5 | 2 | 8.3 | 33 | 52.4 | 0 | 0.0 |
| On treatment* | 63 | 51.2 | 21 | 87.5 | 21 | 33.3 | 21 | 58.3 |
| Referred | 18 | 14.6 | 0 | 0.0 | 3 | 4.8 | 15 | 41.7 |
| Dead | 7 | 5.7 | 1 | 4.2 | 6 | 9.5 | 0 | 0.0 |
| *cases that were still receiving medical care at the time of data reporting | | | | | | | | |
