# Supplementary material for: Second-year sentinel surveillance of bacterial meningitis in Jordan, Egypt, and Iraq: year 2 findings from the MenMap Network (2024–2025)
Source: IJID Reg. 2026 Aug 28;21:100984. doi: 10.1016/j.ijregi.2026.100984 (PMC13672829; doi:10.1016/j.ijregi.2026.100984)
Supplement: Supplementary file 9 [file mmc9.docx]

Table S8: Clinical outcomes among laboratory-confirmed bacterial meningitis cases by detected pathogen, Year 2 (December 2024–November 2025)

| **Case Outcome** | **Pathogen** | | | | | | | |
| --- | --- | --- | --- | --- | --- | --- | --- | --- |
|  | ***S. pneumoniae*** | | ***H. influenzae*** | | ***N. meningitidis*** | | **Total** | |
|  | **n** | **%** | **n** | **%** | **n** | **%** | **N** | **%** |
| Recovered | 27 | 25.2 | 2 | 40.0 | 6 | 54.5 | 35 | 28.5 |
| On treatment* | 55 | 51.5 | 3 | 60.0 | 5 | 45.5 | 63 | 51.2 |
| Referred | 18 | 16.8 | 0 | 0.0 | 0 | 0.0% | 18 | 14.6 |
| Dead | 7 | 6.5 | 0 | 0.0 | 0 | 0.0% | 7 | 5.7 |
| *cases that were still receiving medical care at the time of data reporting | | | | | | | | |
